# Genetic Modifications of Developmental Dyslexia and Its Representation Using In Vivo, In Vitro Model

**DOI:** 10.1055/s-0044-1781456

**Published:** 2024-02-27

**Authors:** Zakiyyah M.M. Zaki, Siti A. Ali, Mazira M. Ghazali, Faidruz A. Jam

**Affiliations:** 1 Department of Neurosciences, School of Medical Sciences, Universiti Sains Malaysia, Kelantan, Malaysia; 2 Department of Electronic Engineering, Faculty of Engineering and Green Technology, Universiti Tunku Abdul Rahman, Kampar, Perak, Malaysia; 3 Centre for Healthcare Science and Technology, Universiti Tunku Abdul Rahman, Kampar, Perak, Malaysia; 4 Faculty of Medicine, Universiti Sultan Zainal Abidin, Terengganu, Malaysia; 5 Department of Biochemistry, Faculty of Medicine, Manipal University College Malaysia, Melaka, Malaysia

**Keywords:** dyslexia, genetic of language disorder, developmental disorder

## Abstract

Dyslexia is a genetic and heritable disorder that has yet to discover the treatment of it, especially at the molecular and drug intervention levels. This review provides an overview of the current findings on the environmental and genetic factors involved in developmental dyslexia. The latest techniques used in diagnosing the disease and macromolecular factors findings may contribute to a higher degree of development in detangling the proper management and treatment for dyslexic individuals. Furthermore, this review tried to put together all the models used in the current dyslexia research for references in future studies that include animal models as well as in vitro models and how the previous research has provided consistent data across many years and regions. Thus, we suggest furthering the studies using an organoid model based on the existing gene polymorphism, pathways, and neuronal function input.

## Introduction

Dyslexia has been indicated since over a hundred years ago, with intensive research done circa the 1950s. However, no massive progression exists in determining the mechanism and its treatment. Meanwhile, the cases have increased over the years. The outcome or burden is not rapidly apparent as in other chronic diseases. Nevertheless, it may cause implications for the human resources burden in the long run. Increasing cases will be reflected in the time increase, and teachers need to concentrate on the dyslexic classes. Multiple factors, including cognitive disabilities, symptomatic, sensorimotor, as well as comorbidities, are used to categorize dyslexia.

Dyslexia diagnosis has been consistent across the globe. Nonetheless, multiple recent reports are suggesting that dyslexia has turned into a spectrum similar to autism as it needs multiple facades for confirmation rather than simply reading and speech problems. Despite being a reading and phonological abnormality, dyslexia is discovered across many languages with different word sounds and meanings. The current gold standard for dyslexia worldwide involves structured literacy (Orton Gillingham). Due to the fact that reading requires clearly taught linguistic components, this therapy course structure is internalized. This covers syllabification, phonology, morphology, as well as encouraging children to automatically use this information for language decoding (reading) and encoding (spelling). This gold standard therapy has been proven to be helpful to many worldwide. However, a recent review published in Hall et al 1 presents how the gold standard may not be a magic bullet to solve all existing dyslexic symptoms in a prolonged interval after years of default from the therapy. Therefore, scientists have been looking for the root cause of the problem molecularly instead of treating it at the endpoint of the symptoms to fight this chronic disability that seems to grow massively in decades. Hence, this likely shared problem could arise from the genetic and yet to be fully discovered molecular dysfunction. Addressing this issue requires tireless efforts in determining the factors involved in developmental dyslexia (DD) to precisely recognize the individuals and indirectly provide initiative for better preventive majors. Many researchers have tried to look at the clinical perspectives. Nonetheless, the lack of data on the biomolecular production of this disorder provides a stepping stone for this review to gather the existing data, although not close to thoroughly, on genetic modification in dyslexia cases and its importance.

## Dyslexia Phenotypes

Dyslexia phenotypes refer to the various subtypes or categories of dyslexia, each with unique characteristics and symptoms. Phonological dyslexia, rapid naming deficit dyslexia, surface dyslexia, attentional dyslexia, and motor dyslexia are the most common groupings of dyslexia individuals reported worldwide. Here, phonological dyslexia refers to difficulty in decoding and sounding out words.

In phonological dyslexia, all children start with phonological struggles until a certain period. They also struggles to attain the phonetics sound of the alphabet. According to Wolf and Bowers, phonological dyslexia does not only involve the full inability to provide the correct alphabet sound but also the delayed response to it most of the time, and they are incorrect. 2 Hence, it is considered double deficit dyslexia that obstructs time and fluency and is closely related to the rapid naming deficit types. 3 Other types of dyslexia include surface dyslexia, attentional dyslexia, and motor dyslexia, which varies in the symptoms presentations. When it comes to surface dyslexia, this condition is characterized by difficulties spelling and trouble in whole-word recognition by sight. It is frequently linked to semantic dementia. 4

Generally, the diagnosis of dyslexic children involves deficits in learning abilities, mainly reading and speech disturbances. Recent studies are expanding on the involvement of other neuropsychological and academic evaluations through various modalities, including neuroimaging techniques. Neuroimaging studies in dyslexia have also revealed variations in the signal transmission efficiency in white matter tracts, cortical gyrification, gray or white matter volume, as well as multiple brain lobes in the left hemisphere. 5 6 These findings go beyond patterns of brain activation. In fact, the earliest findings of postmortem studies for individuals with severe reading abilities were found to have cortical anomalies, 7 although the case report was doubtfully specific to DD as it was accompanied with nocturnal seizures, which rarely sighted in many dyslexic cases.

The theory of magnocellular neurons in DD is recently highlighted, considering the presence of auditory and vision problems in DD. 8 The main disturbance in DD is not the inability to read. However, it stems from the inability of hearing and vision to differentiate the phonemes between letters. The relation between these two senses leads to magnocellular neuron involvement as these types of cells originate from the same source of neural tubes during embryonic development. 9 Most cases of dyslexia too indicating shared autoimmune disorders are evidence of the problem rooted way deeper in the molecular function of the brain. 8 Common shared phenotypes from neuroimaging and inflammatory studies suggest the hidden molecular root causal factor of this disorder.

Children who have family members with dyslexia are more likely to develop the disorder, with the likelihood increasing by 40 to 60%. This is according to a study on children with familial high risk of dyslexia that indicated a pattern of genetic inheritance across three family generations. 10 Besides, a study on twins have presented that genetic factors significantly contribute to the familial clustering of dyslexia. 11 12 Note that epigenetic effects on dyslexia were seen in some children who inherited learning disabilities from their parents but not in others. In fact, some of them can reach normal learning milestones despite having the same chance of inherited dyslexia. Those who have normal learning milestones are classified as compensated (diagnosed with dyslexia but with normal learning abilities) or possibly carriers (no learning difficulties at all but with affected family members). The only reasons that differentiate these three heterogeneities of phenotypes in familial dyslexia risk could be caused by the environmental factors that cause the expression of the specific mutated gene to be differentially expressed.

A meta-analysis comparing the development of language and decoding skills in three groups of preschool children (dyslexia with familial risk [FR], normal reader with FR, and healthy control group with no FR) indicated that the first group had the most severe impairments in language development, followed by the second and the control group. 13 From this analysis, it could be derived that genetic inheritance in familial history impacts children's learning abilities. However, the interplay of different environmental factors for the children being brought up might differ from the other two groups, who might have well-educated families, well nutrition, and a better communication environment, which could be lacking in some children with FR. This is where epigenetic studies can be employed to explain the heterogeneity of these different phenotypes.

The heterogeneity of phenotypes in dyslexia can be categorized into two: (1) learning difficulties in reading, writing, or spelling, (2) heterogeneity of brain connectivity, structures, and functions. As for the first heterogeneity of phenotypes, it is generally observable and has already been briefly discussed above. Nevertheless, the latter is rarely discussed in detail concerning the genetic influence on the different activities of the brain. Abnormal brain activity begins as early as the prereading age, between 2 and 5 years old with recent findings reporting that prereading children with FR had substantial gray matter volume in the left fusiform gyrus, left occipitotemporal, right lingual gyrus, and bilateral parietotemporal. 14 These brain areas were positively correlated with the ability of rapid automatized naming, grapheme–phonemic decoding, and phonological processing. Consequently, the same brain regions were also hypoactivated in another study of prereading children with FR compared with the non-FR children, indicating general brain alteration across prereading age children. 14 Interestingly, neural activity started to be altered differently when the children reached primary school age. At this age, the brain starts compensating for any neural abnormality by lateralized brain activity similar to healthy non-FR children. 15 A high temporal resolution event-related potential study by van Setten et al 15 presented prominent early emergence of neural peak component N1 involved in presensory processing of visual and phonological tasks in children with FR as compared with non-FR children with no significant differences of hemisphere lateralization between these groups. Instead, it had lateralized activity to the right hemisphere similar to the non-FR group, 15 uncommonly seen in children with FR. 14 16 17 Table 1 below briefly lists some literature on brain neuroimaging studies in children with FR and non-FR of dyslexia. Based on the literature, the brain abnormality in structural activity and functioning identified in early prereading children with FR of dyslexia proposes that the brain changes and alterations may possibly present since birth and develop in reading age. The compensatory effect occurs when reaching a certain age of life. However, this opinion is yet to be conclusive, as other determinant factors, such as environmental factors, may affect different individuals differently.

**Table 1:** Brain activity abnormality and the neuroimaging studies in dyslexia patients with familial risk

Neuroimaging techniques	Subjects	Abnormal brain region/source activity	Findings summary	Source
Event-related potential (ERP)	● Three study groups (18 with low familial risk (FR) without dyslexia, 15 with high FR without dyslexia, and 12 with high FR with dyslexia ● Mean (age): 12 y old	All research groups saw somewhat earlier N1 ERP component emergence, with no discernible variations in the lateralization of the brain hemisphere	The absence of left lateralization, commonly observed in most dyslexia studies, could be due to the effect of the different experimental paradigms used and the possibility for neural compensatory effects the children have been exposed to in the learning environment at school	15
Functional magnetic resonance imaging (fMRI)	● 14 pre-children with FR of dyslexia (not yet diagnosed with dyslexia) and 14 non-FR ● Mean (age): 5.6 y old	Hypoactivation in the left hemispheric prefrontal during rapid auditory processing (RAP)	Similar hypoactivation brain region to older children (already diagnosed with dyslexia), suggesting for alteration of RAP and phonological processing since the early prereading phase, which could be one of the explanations for struggling in mentally linking to the association of graphene and its phonemic sounds (grapheme–phoneme conversion)	16
	● 35 infants with FR and 63 infants with non-FR ● Mean (age): 8.5 mo old	Between newborns at family risk vs. infants not at FR, there is a significant difference in the functional connectivity of the left fusiform gyrus	In this study, only the left fusiform gyrus was observed as having abnormal functional connectivity. Hence, this suggests that the functional brain area affected or altered earliest after birth in infants with FR is the left fusiform gyrus	17
Magnetic resonance imaging (MRI)	● 10 prereading children with FR and 10 healthy non-FR ● Mean (age): 5 y and 11 mo	Significant reduction of gray matter volume in children with FR in left occipitotemporal, bilateral temporoparietal, left fusiform, and right lingual gyrus	Brain structure abnormality possibly develops at birth and early childhood	14

## Dyslexia Heritability and Epigenetics Factor

In contrast to the previous belief that dyslexia is a monogenetic disorder, it is currently strong evidence that it evolved according to environmental influence. Interestingly enough, replicable gene modifications are seen across races and nations, suggesting that there is a gene responsible for the generation of language and reading disabilities.

According to popular belief, language disorders are brought on by the monogenic inheritance of uncommon variations, in which only one gene can disrupt function through recessive or dominant heredity. The term “epigenetics” refers to the dynamic molecular modifications that are deposited on chromatin within a cell's nucleus and that influence the regulation of DNA-related processes, including chromatin organization, DNA repair, RNA transcription, as well as splicing. This causes the altered gene to remain in the genome and be passed to the offspring, driven by the vast environmental factors. The relation of genetic modification across the generations in the development of dyslexia should not go unnoticed.

The effects of environmental factors on epigenetics are also possible for dyslexics without a familial history of dyslexia. An early concept suggests that dyslexia is caused by the brain's compensating mechanism for the corticolimbic systems' reactivation after being activated by high levels of stress. This practice can lessen neuroplasticity, which is essential for learning. 18 The hypothalamic–pituitary–adrenal axis is claimed to be consumed by Early Life Stress, which is thought to be epigenetically controlled upon DD in childhood. Other than that, chronic exposure to stress hormones causes the brain to adapt, which alters the structure of the brain and causes the development of cortisol resistance. 19 Additionally, exposure to a home literacy environment predicts literacy readiness in children. Home literacy environments refer to active language interaction between parents and children, that is, shared reading and teaching diverse vocabulary and complex linguistic structures (syntax), which are highly correlated to children's literacy development. 20 Here, stronger reading literacy emerged in children with familial dyslexia risk exposed to a home literacy environment. 21 Moreover, pregnancy lifestyle is also considered a risk factor for dyslexia development. Any uneventful event, such as preterm delivery, causes immaturity in brain development and disrupts white matter brain organization in the learning brain areas. 22 23 Table 1 summarizes the environmental risk factors associated with a high risk of epigenetically induced changes and susceptibility to dyslexia.

Both dyslexia phenotypic variability was thought to result from several epigenome interactions that were controlled by environmental variables. 19 Environmental factors include high-stress levels, genetic variations, cognitive development, parental education or background, socioeconomic status, home literacy environment, health problems, and maternal diet during pregnancy. All these factors could interact abnormally with an individual's genomic structure, resulting in epigenetic alterations. Due to that, not every dyslexic has similar characteristics. For example, some may have had difficulty with sound encoding, some may have been good at it but had a poor attention span, and some may be good at both skills but have difficulty expressing the sound–grapheme relationship. On top of that, some may have behavioral abnormalities. Hence, dyslexia characteristics are regarded as unique to individuals and have a wide spectrum of characteristics, but the domain area of abnormalities is generally under the scope of learning difficulties. The epigenetic study of dyslexia is still in its infancy, considering that limited human studies have been done on it. Thus, fundamental studies connecting the epigenetics of dyslexics with familial history and environmental factors are limited. Table 2 summarizes the environmental risk factors associated with a high risk of epigenetically induced changes and susceptibility to dyslexia ( Table 2 ).

**Table 2:** Summary of the literature findings on environmental factors to epigenetic susceptibility in dyslexia

**Environmental factor**	**Pathophysiology**	**Source**
Psychological stress	High-stress levels induce overactivation of the hypothalamic–pituitary–adrenal stress axis, which potentially reduces: ● The development of learning brain areas, specifically the prefrontal cortex ● Reduces levels of gene regulatory factors involved in neuroplasticity and neural maturation (e.g., brain-derived neurotrophic factor and transcription factor EB) ● (iii) Disrupts neural network connections in the amygdala, insular cortex, and hippocampus, all involved in cognitive function	18
Maternal lifestyle during perinatal and pregnancy (maternal diseases, malnutrition, unfavorable events. For example, preterm delivery)	Disrupt normal brain development by reducing the organization of white matter in the left superior longitudinal fasciculus (connects frontal and temporal language areas), which is essential in processing specific skill development such as phonological awareness and word decoding	22 23
Home literacy environment (parental education, socioeconomic status)	● Repeated exposure from an early age to active language interaction between parents and children builds a foundation of decoding skills and phonological awareness ● The intrinsic motivation to read may significantly rise as a result of literacy exposure	21

## Neurobiology of Dyslexia

### Brain Anatomy and Dyslexia

The brain volume of those with dyslexia and reading disability was significantly reduced in the right precentral gyrus when reading levels matched those of the control groups. However, in an age-matched group, the dyslexia individuals are significantly reduced compared with the controls in multiple areas, namely left cingulate, right middle frontal gyrus, left middle temporal gyrus, right superior temporal gyrus, as well as right precentral gyrus in the gray matter area. At the same time, the white matter volume demonstrates substantial shrinking in the case group at most frontal and central gyri, central, and thalamus lobule. 24 25 In addition, the left superior temporal, left insula, as well as subcortical areas in DD also exhibited signs of reduced gray matter volume and cortical thickness overlapping, according to Kujala et al in 2021. 26 These alterations are associated with lower reading and phonological test scores. 27

### Genetics of Dyslexia

Recognizing a specific word and the ability to read it requires an orchestra of linguistic, visual, and attentional regulations. The anatomical and functional structure of the brain throughout development greatly influences these results. Various anomalies were observed in dyslexia, including a reduction or increase in the left temporal, parietal, and fusiform firings to identify the alphabets, words, meanings, and phonic sounds, as well as an inability to discriminate between simulations and activations.

#### KIAA0319

The cellular neurobiology of reading disabilities was further elaborated using animal models of the reported genes, namely the KIAA319 knockout (KO) mouse. Promising results were shown with abnormalities in the auditory temporal processing with consistent anatomical changes in human imaging data. 28 29 The model of hemispheric asymmetries proposed by Brandler–Paracchini 30 assumes that specific genes are involved in the division and development of brain midline structures affecting the ciliogenesis and overall reading ability or language lateralization. KIAA0319 gene first four exons are highly associated with dyslexia with a single-nucleotide polymorphism (SNP) specifically at *rs17243157* G/A was significantly associated with left-lateralization activation during development of the posterior superior temporal sulcus. 31 Consistent with psychological view of handedness influences the language lateralization and spatial attention by cortical dominance. 32 Although it is not clear how the methylation and polymorphism directly aggregate to the variation in reading disability, the mechanisms could involve the neuronal migration, with reduced midsagittal corpus callosum volume and impaired auditory stimuli processing that could collectively cause slow respond and naming disability or consonant confusion in the dyslexic individuals.

#### DYX1C1

A report in 2013 concentrated in Italy that studied 165 nuclear families with at least one member with DD proposed the involvement of candidate genes *DYX1C1* that was strongly associated with the status of maternal smoking habits during pregnancy, birth weight, and socioeconomic status. While all the phenotypes do not clearly define the environmental influence nor epigenetics involvements, the presence of consistent traits among the affected children and adults may contribute to the alteration of the multiple candidate genes across generations. 33 The first four exons of *KIAA0319* gene were reported to be associated with dyslexia with evidence of DNA methylation in its promoter region are able to predict the attentional modulation of language lateralization through listening task specifically in male. 32

Nine risk loci (DYX1–DYX9, where DYX stands for dyslexia) have been associated with dyslexia, albeit not all researchers have been able to duplicate these findings. 34 35 Six potential genes have been found at certain of the nine repeated risk loci using more accurate mapping techniques, a risk locus is identified by its chromosomal number on one of the 23 human chromosomes and by the length of its two arms, either short (p) or long (q). The six potential genes are located on chromosomes 15q21, 6p21, 2p16-p15, and 3p12-q12. These genes are *DYX1C1* in the DYX1 locus, *DCDC2* and *KIAA0319* in the DYX2 locus, *C2Orf3* and *MRPL19* in the DYX3 locus, as well as *ROBO1* in the DYX5 locus. The effects of *DYX1C1* , *DCDC2* , *KIAA0319* , and *ROBO1* on prenatal processes of brain development have been studied, particularly their role in neuronal migration—the movement of immature neurons from the location where they initially develop to their final position in the brain. The first investigation of their function in brain development in rodents found that they also played a role in the emergence of links once neurons arrive at their endpoint, for example, neurite—axon and dendrite—outgrowth and guidance. 36 A family or network of genes that communicate with one another via molecular signals controls these two early brain development phases genetically in general. In the past decade, animal-based models have been established to fully reveal the mechanism involved up to its anatomical and behavioral phenotypes. Based on Table 3 , there are multiple population-based studies reported. Although replicable in the genotypes, the discrepancy between the research may be caused by mutations in dyslexia that are in noncoding areas that influence how structural genes express themselves, minutely affecting the expression of proteins. 33 37 Genetic involvement in dyslexia and generally reading disabilities have recently been the focus. Note that the latest discovery of 42 genome-wide-significant loci linked to it, 38 thus strengthening the genetic involvement in DD. Furthermore, they are mainly categorized as 15 genes linked to cognitive ability, whereas the remaining 27 genes are new and undiscovered, potentially very specific for dyslexic cases. The multiple genetic predispositions from this study alone reflect the axonal guidance abnormality in most dyslexic cases.

**Table 3:** Multiple population-based data from Australia (Eising et al 2022; Paracchini et al 2010), India (Venkatesh et al 2014), United Kingdom (Gialluisi et al 2021), Finland (Eising et al 2022), and Germany (Leibig et al 2020) have shown that there is strong and repetitive evidence in the involvement of DYX1C1, DCDC2, KIAA319, and ROBO1 gene single-nucleotide polymorphism

Population ( *N* )	Polymorphism	Gene	Publication and year
Austria, Germany, Switzerland, Finland, France, the Netherlands, Hungary, the USA, Spain, the UK, Canada, and Australia (34,000)	SNP rs11208009 C/T on chromosome 1	DOCK7 ANGPTL3	58
India (210)	SNP rs12899331 on chromosome 15	DYX1C1	59
United Kingdom (2,274)	SNP rs6035856 on chromosome 20	LOC388780 (No single variant association)	60
Chinese (115)	SNP rs4535189 and rs6803202 on chromosome 3	ROBO1	61
Australia (2,868)	SNP rs804075 on chromosome 15	DYX1C1	62
German (93)	SNP rs6935076 on chromosome 6	KIAA0319	29

Abbreviation: SNP, single-nucleotide polymorphism.

Notes: A recent finding suggests that there are newly validated 13 loci and ten existing loci associated with dyslexia in Chinese and European population studies. Dyslexia candidate genes (DCGs) have been reported with over 50 genes probably involved in the population-based data.

Putting all the genes together in finding the pathway that may be affected in the brain of dyslexic indicates that they are susceptible to neurite growth, neuronal migration, ciliary structure, cortical morphogenesis, as well as function. 33 36 Hence, a huge variation in how genes contribute to brain function presents that the neurobiology of dyslexia comprises a massive alteration to the vast network of the brain rather than point mutation.

## Experimental Models from Existing Genetic Data

### Animal Models

Reading and comprehension disability is a specific phenotype intrinsic to human physiology. Therefore, searching for translatable mechanisms in the animal model has been done for decades. Recent neuroimaging studies has established how the brain activity in dyslexics differ compared with normal individuals. Thus, further studies extends to animal models, particularly mouse models as it is less laborious in terms of handling and manipulation. Although research using macaque has been widely employed as it represents more of the human brain than other species, there are unfortunately no available reports using this model yet. This is perhaps due to the seemingly inconclusive reports from the mouse and clinical reports. Other than that, the resulting animal model's phenotypes shall bear a reasonable resemblance to the human counterpart. A recent review by Galaburda in 2022 summarizes the animal model for dyslexia disability. 39 However, not only that the animal modeling consolidated with the presence of cerebral lateralization, but also the cerebrocortical dysfunction models have been proposed.

The big data indicate multiple reports consistently find the exact polymorphism in the *KIAA319* gene. Thus, manipulating this gene may provide a significant view of the mechanism involved in dyslexic individuals. *KIAA319* KO demonstrates the impact of this gene in neuronal migration in zebrafish models in the brain and eyes. However, it is not specific as it is somehow expressed in other organs of the fish as well. On the other hand, Guidi et al generated a KO mouse model with another homolog, *KIAA0319L* . 40 The results contradict this model, as no evidence of neuronal migration defects exists. Nevertheless, the phenotypes displayed abnormal auditory processing when tested with Auditory Brainstem Response test. A more precise and recent behavioral test by Perrino et al 28 presents that the *KIAA0319* KO mouse reacted differently to the altered prepulse inhibition test, whereby the KO mouse performed significantly worse at short acoustic gap auditory processing.

*DCDC2* gene in vivo analysis shows that the knockdown of this gene causes impairment to the visuospatial performance in mice. 41 This further being translated in a clinical model where young children (kindergarteners) with reading disability performs poorly in the virtual maze learning task, having a greater risk of inconsistency in these parameters seen in those with a genetic microdeletion in *DCDC2* gene. 42 Interestingly, the mutation does not cause anatomical deficits in human and mouse models. Currently, the proposed therapy for dyslexic children is vision therapy, as there are multiple reports of dyslexic eyes unable to focus on the alphabet and blurred and double visions. Therefore, this finding is crucial as it connects the dots and proposes the potential of the *DCDC2* KO mouse model to be the standard for treatment and therapy for dyslexia cases.

The next candidate gene, the *ROBO1* gene, is familiar to be an axon guidance receptor regulating the connection between brain hemispheres. In particular, it involves signal processing from one side of the brain to another as well as the interaction of interneurons for migration in the forebrain area. 43 While it is not uncommon to have hearing troubles in dyslexic cases, this may eventually be part of the mechanistic pathway in auditory dyslexic cases.

The most recent study by Price et al in 2022 hypothesized that the presence of dyslexia (identified as a reading disability) was prominently linked to gene set data for Neuronal Migration and Axonal Guidance. 44 This is intriguing as there are reports of autopsy brains of dyslexics showing neuronal heterotopias and cortical dysplasia. To answer such linkage, researchers postulate that dyslexia occurs before the child is born and at the embryonic level. This is because cortical dysplasia usually happens when the developing brain cells fail to reach and migrate to the area they are genetically decided upon before birth. Note that neuronal stem cells at this stage are flexible and responsive toward the surrounding environments. After birth, the neuronal stem cells can no longer travel across the brain as their multipotency has reached maturation levels. This eventually causes birth defects, albeit not physically presented, but phenotypically prominent in the children. 33 Some of the most studied genes in dyslexia with replicable data across the globe are listed in Table 4 .

**Table 4:** The replicable candidate genes in dyslexia reported using clinical samples collected across various countries and its functions tested using in vivo animal models

Genetic factor	Mechanism of action	Source
*DYX1C1*	● An axon guidance receptor gene with a missense mutation mouse resembles primary ciliary dyskinesia phenotypes, and expanded ventricles are presumed as hydrocephalus ● Hormonal control of neurite extensions through estrogen receptor interaction in the rat hippocampal region	63 64
*ROBO1*	● Regulation of axon guidance receptors connecting between two hemispheres; corpus callosum formation ● Neuronal migration during development to form dendritic spines for correct terminal positioning of neurons	43 65 66
*DCDC2*	● Alteration of gray matter development and temporal–parietal white matter composition involving neocortical migration	67 68
*KIAA319* *KIAA0319L* (Homologous)	● “Signature” molecule production on the surface of nerve cells is controlled. These aid in regulating how they migrate to take up their ultimate places throughout the in utero development of the unborn brain ● Through the opposing switch of Pax6 and Sox10, cell cycle maintenance occurs when the human neuroepithelium develops into neural progenitor cells ● Severe volumetric losses and a shift toward fewer big and smaller neurons in the medial geniculate nucleus, as well as severe auditory processing deficits related to rapid/brief stimuli, are also seen	28 48 69

These reports present that the main anomalies attained by human dyslexia cases are focused on neuronal migration and ciliary formation. The left–right asymmetry planum in vertebrates is controlled by the ciliary body in the developing brain, according to research that supports this. For a clearer illustration of the condition, the best animal model of dyslexia should be matched to these two abnormalities. 45

### In Vitro Models

The intricacy of reading or crucial abilities like decoding, phonological awareness, or orthographic coding could not be studied in animal models due to this constraint. Thus, an in vitro technique employing particular human neurons and tissues has been carried out in order to give a mechanism to link results to brain areas important for reading. 46 47 Furthermore, we have summarized several in vitro approaches in Table 5 developed to model dyslexia disorder and understand the molecular mechanism involved in this disorder.

**Table 5:** Summary of the cell lines used in previous dyslexia studies

Cell lines	Gene(s) investigated	Source
Human embryonic stem cells (hESCs)	KIAA0319	48
Retinal pigment epithelial cells (RPE1)	KIAA0319	49
HEK293T and HeLa	KIAA0319	50
Human retinal pigmented epithelial cell line immortalized with hTERT (hTERT-RPE1)	Dyslexia-associated genes (DYX1C1, DCDC2, and KIAA0319)	51
Human long-term self-renewing neuroepithelial stem (NES) cells	Ciliary genes (DYX1C1)	52
Hippocampal and cortical neuron cultures prepared from the brains of E17 rat embryos	DCDC2	54
Human neuroblastoma SH-SY5Y cells	DYX1C1	53
Neuro-2a, hippocampus neurons prepared from E18 Sprague Dawley rat embryos, HEK293T, and COS7	DOCK4	55
HEK293 cells and Epstein–Barr virus (EBV)-transformed lymphoblast cell lines from the DYX5-linked family and controls	ROBO1	56

The *KIAA0319* gene encoded in the DYX2 locus on human chromosome 6p22 possesses an important role in neuronal differentiation that provides the foundation for higher cognitive function. A study done by Paniagua et al demonstrates that this gene is critical for neuronal differentiation. They employed the human embryonic stem cells (hESCs) model of cortical neural differentiation and examined the *KIAA0319* function during neurogenesis. By employing CRISPRi/dCas9-KRAB in H7dCas9 − KRAB-b6 hESCs focusing the transcription start site, they showed neuroepithelial cell differentiation is affected in the KIAA0319 knocked down cells. 48

The next step was employing CRISPR-Cas9n to examine the function of the KIAA0319 gene in cilia formation and cell migration in RPE1 retinal pigment epithelial cells. With the *KIAA0319* KO model, cilia length improved along with cell migration as well as force exertion. 49 The surface expression of *KIAA0319* has formerly been shown to be controlled by endocytosis employing HEK293T and HeLa cells. 50 Hence, this finding supports the notion that internalization and recycling of the protein may be involved in fine-tuning its role in neuronal migration.

Utilizing the human retinal pigmented epithelial cell line immortalized with hTERT (hTERT-RPE1), the involvement of dyslexia candidate genes (DCG) like *DYX1C1* , *DCDC2* , and *KIAA0319* in ciliary function have been explored in prior research of the same type. The research also identified functional noncoding elements, called X-box promoter motifs, in DCG promoter regions, which can be focused on mutation screening in dyslexia as well as ciliopathies related to these genes. 51

In another study, the function of ciliary genes in developing neural cells was examined using human long-term self-renewing neuroepithelial stem (NES) cells. 52 Here, human induced pluripotent stem cell (iPSC)-derived NES cells imitate in vitro human neural development. Since they resemble neuroepithelial cells in vivo, self-renew in the presence of fibroblast growth factor as well as epidermal growth factor, and are capable of differentiation into neuronal and glial cells, this cell line is the ideal model for neurodevelopmental processes and diseases. This research demonstrated that cilia-related genes, particularly those connected to ciliopathies with neurodevelopmental deviations, were highly favored among upregulated genes during differentiation. Furthermore, it verified that primary cilia existed throughout neuronal differentiation. Concentrating on DCGs, RNA sequencing identified 33 of 50 DCGs in NES cells, and seven candidate genes, which include *DYX1C1* (DNAAF4), showed upregulation during differentiation to neurons. Lastly, the results indicated that ciliary genes play a role in neuronal cell differentiation and demonstrate that NES cells are a useful human neuronal model for studying possible ciliary as well as dyslexia genes.

*DYX1C1* is the first gene identified for dyslexia susceptibility. Previous studies have verified the role of *DYX1C1* in controlling neuronal migration throughout embryogenesis as well as learning in rodents. Therefore, understanding the control of *DYX1C1* and the potential functional significance of genetic variation in the promoter of *DYX1C1* were the goals of an investigation conducted by Tapia-Páez et al in 2008 53 on human neuroblastoma SH-SY5Y cells. They represented three possibly functional SNPs in the promoter of *DYX1C1* and implicated TFII-I, PARP1, and SFPQ as the three transcription factors for *DYX1C1* .

In primary rat hippocampus neurons, *DCDC2* localizes to the main cilium and is located near the ciliary kinesin-2 component Kif3a. Hippocampal and cortical neuron cultures generated from the brains of E17 rat embryos were employed for functional investigations of *DCDC2* using overexpression and knockdown experiments. It was evident from the experiment that *DCDC2* has a role in the structure and function of primary cilia through Shh and Wnt signaling. 54

Another associated gene with dyslexia is *DOCK4* . The growth of neurons and social behaviors are both influenced by this gene. Huang et al 55 demonstrated that mutant Dock4 has a reduced capacity to activate Rac1 and Rap1 using HEK293T cells. In this work, they discovered Dock4 mutants showed impaired function in promoting neurite outgrowth as well as dendritic spine formation utilizing Neuro-2a cells and hippocampal neurons obtained from E18 Sprague Dawley rat embryos as models. Besides, COS7 cells were used to investigate cell morphology as well as the cytoskeleton, where Dock4 mutants showed disruption in their actin cytoskeleton.

*ROBO1* has an important developmental role and was found to be dysregulated in dyslexia disorder sufferers. Massinen et al 56 devised an investigation to define variation within the susceptibility haplotype in an attempt to identify variants that could shed light on the regulatory effects underlying the dysregulation of *ROBO1* as the molecular mechanism for the suppressed expression of *ROBO1* from the DD susceptibility haplotype is unidentified. They discovered that *LHX2* controls *ROBO1* in humans using HEK293 cells, Epstein–Barr virus-transformed lymphoblast cell lines from the *DYX5* -linked family, including controls.

The most recent findings on the usage of human fibroblasts induced into iPSCs and grow in the culture as an organoid has caught attention of many. Considering the difficulty of getting the correct representation of animal models for language disorders, the utilization of brain organoid becomes a central focus in these studies.

As of the now, there are more than 50 models of human brain organoids and assembloids carrying specific gene mutation and phenotypes ranging from germline to karyotypic defects and mosaic diseases 57 that could lead to a better intervention for personalized medicine and efficient drugs delivery to the brain.

## Conclusion

Throughout the years, dyslexia has not been regarded as a disease but a result of different thinking and learning styles. Hence, treatment management has been focused on educational counseling or tutoring, thus eliminating the symptoms. However, using the evidence from multiple research works, there is a consolidating factor that may cause the inability and difference in the brain development of dyslexia that lead to a prolonged disability even in later years of life. Therefore, it is important to address such issues for the better future of dyslexic individuals.

In a more stratified view, there are still little to no data on the Southeast Asia cases of dyslexia nor reports on the genetic basis of such population. This review shall provide a basis for a genome-wide association study for other developing countries to provide to the currently existing big data, making it more comprehensive as the outlook has been centrally focused on European with little information on Asian. Other than that, this could contribute to the power of the gene of interest in previous studies or possibly bring more interesting data to the existing ones.

This sharing of genetic modifications could be a window to the genetic basis of dyslexia and its mechanism, aiding in generations of treatment management and counseling and tutoring aids over the years. The study model in dyslexia is normally used to develop a drug treatment for the disease. Nonetheless, it is limited as this is specifically involved in reading ability, which is never seen in any other animal models. Despite the challenges, many scientists manipulated the sound ability of animals for such purposes. However, it is difficult to pinpoint the exact similarities between the reading system and the whole sound system in the brain, albeit the network connections. New research methods propose the usage of brain organoids on multiple cumulative data on gene alteration in dyslexic brains. This, nevertheless, may take a couple of decades to be turned into reality.
